# Histone Deacetylase Inhibition Attenuates Aortic Remodeling in Rats under Pressure Overload

**DOI:** 10.1155/2020/4705615

**Published:** 2020-07-24

**Authors:** Hanna Jung, Eunjo Lee, Inkyeom Kim, Gun Jik Kim

**Affiliations:** ^1^Department of Thoracic and Cardiovascular Surgery, Kyungpook National University Hospital, School of Medicine, Kyungpook National University, Daegu 41944, Republic of Korea; ^2^Bio-Medical Research Institute, Kyungpook National University Hospital, Daegu 41944, Republic of Korea; ^3^Department of Pharmacology, School of Medicine, Kyungpook National University, Daegu 41944, Republic of Korea; ^4^Cardiovascular Research Institute, Kyungpook National University, Daegu 41944, Republic of Korea; ^5^PBK21 PLUS KNU Biomedical Convergence Program, School of Medicine, Kyungpook National University, Daegu 41944, Republic of Korea

## Abstract

The use of histone deacetylase (HDAC) inhibitor is a novel therapeutic strategy for cardiovascular disease. Studies have shown that many HDAC inhibitors have the ability to reduce the aortic remodeling in various animal models. We hypothesized that the HDAC inhibitor, MGCD0103 (MGCD), attenuates aortic remodeling in rats under pressure overload-induced by transverse aortic constriction (TAC). The aortic ring tension analysis was conducted using the thoracic aorta. Sections of the aorta were visualized after hematoxylin and eosin, trichrome, and Verhoeff-van Gieson staining, and immunohistochemistry. The expression of genes related to aortic remodeling (*αSMA*, *Mmp2*, and *Mmp9*) and angiotensin receptors (*Agtr1* and *Agtr2*) was determined by quantitative real-time polymerase chain reaction. There was a significant decrease in relaxation of the aorta when treated with MGCD. Fibrosis of the aortic wall and expression of angiotensin receptors increased in TAC rats, which was attenuated by MGCD. These results indicate that MGCD, an HDAC inhibitor, attenuates aortic remodeling in rats with TAC-induced pressure overload rats and may serve as a potential therapeutic target of antiaortic remodeling in pressure overload-induced hypertension-related diseases.

## 1. Introduction

Thoracic aortic diseases such as thoracic aortic aneurysms and aortic dissections are rarely diagnosed until symptoms appear, at which point patients risk a fatal aortic rupture [1]. Hypertension is well known as the most common risk factor for thoracic aortic diseases, but it is still not fully understood how increased pressure on the ascending aorta leads to aortic remodeling [2].

The current clinically used antihypertensive medications are diuretics, which flush the body of excess sodium and water; beta-blockers, which reduce heart rate and workload; angiotensin-converting enzyme inhibitors and angiotensin II receptor blockers, which regulate the renin-angiotensin system; and calcium channel blockers, which relax smooth muscle cells. However, many studies express concern that the current medications seem to drop the systemic blood pressure to a normal range, but do not return the remodeled great vessels or heart to a healthy or normal state; this highlights the need for alternative or additional antiaortic remodeling agents [3].

Beta-blockers have traditionally been the treatment of choice for thoracic aortic diseases as they alter blood pressure. The angiotensin II receptor blocker losartan helps reduce the rate of aortic aneurysm expansion [4, 5]. Since angiotensin II can trigger aortic aneurysm in mice, angiotensin II signaling has a role in aortic aneurysm formation, and several aortic remodeling animal models have demonstrated the importance of angiotensin II receptors in angiotensin II signaling [6–8].

The use of histone deacetylase (HDAC) inhibitor, a promising anticancer drug, is also a novel therapeutic strategy for cardiovascular disease. Studies have shown that various HDAC inhibitors have the ability to remodel the aorta of various animal models. The pan-HDAC inhibitors, trichostatin A and suberoylanilide hydroxamic acid, partly relax the mouse aorta [9]. MS-275 (a class I HDAC inhibitor) and MC-1568 (a class IIa inhibitor) reduce the incidence and severity of abdominal aortic aneurysm and limited aneurysmal expansion in angiotensin II-infused apolipoprotein E-deficient mice [10]. LMK235 (class I inhibitor and HDAC6-preferential HDAC inhibitor) attenuates vascular constriction and aortic remodeling in angiotensin II-infused mice and spontaneously hypertensive rats [11]. MGCD0103 (MGCD), also known as mocetinostat, is a chemically synthesized aminobenzamide class I (HDAC1,2,3) and IV (HDAC11) HDAC inhibitor that mainly inhibits HDAC 1 and is orally available. Several studies have shown its antifibrotic properties [12, 13]. MGCD reverses cardiac fibrosis in rats with postmyocardial infarction heart failure [14, 15] and angiotensin II-infused mice [3].

Transverse aortic constriction (TAC) model is a well-established animal model that demonstrates pressure overload cardiac hypertrophy by inducing left ventricle (LV) hypertrophy through the sudden onset of systemic hypertension [16]. It can also be used as an animal model for thoracic aortic remodeling. Thoracic aortic disease studies mainly use angiotensin II infusion or Marfan syndrome mouse models; however, we used the TAC model in this study as it can investigate the systemic hypertension effect directly on the ascending aorta without pharmacologically or gene mutation-induced factors. Vascular fibrosis is characterized by accumulated collagen, fibronectin, and other extracellular matrix components in the vessel wall and is an important aspect of the aortic remodeling of extracellular matrix in hypertension [17].

HDAC inhibitors have shown cardiovascular protective effects on cardiovascular diseases in animal models. Therefore, we hypothesized that the HDAC inhibitor, MGCD, attenuates aortic remodeling in rats under pressure overload-induced by TAC.

## 2. Materials and Methods

### 2.1. Animals

All institutional and national guidelines for the care and use of laboratory animals were followed and approved by the appropriate institutional committees at Kyungpook National University.

Male Sprague-Dawley rats were housed in a cage at 20–23°C with 12 h light/dark cycles. Minimally invasive TAC was performed using the following protocol [18]. Eleven-week-old rats, weighing 350–400 g, were placed in the supine position and anesthetized with a mixture of ketamine (150 mg/kg, intraperitoneally; Yuhan, Seoul, Republic of Korea) and xylazine (18 mg/kg, intraperitoneally; Bayer, Seoul, Republic of Korea). Under sterile conditions, with surgical loupes and a headlight, partial sternotomy was carefully performed without ripping the mediastinal pleura. The transverse aorta was tied with a 5-0 silk suture using a 22-gauge needle between the innominate artery and left common carotid artery. The needle was then immediately removed, forming a constricted transverse aorta. Sham-operated (sham) rats were subjected to the same procedure without tying the transverse aorta [19, 20]. After the TAC and sham operations, the sternum was closed, and the rats were allowed to recover overnight before being randomly assigned to one of the following four groups: sham (*n* = 8), sham plus MGCD (*n* = 8), TAC (*n* = 8), and TAC plus MGCD (*n* = 8). Precisely, 10 mg/kg of MGCD was dissolved in dimethyl sulfoxide and intraperitoneally injected every other day for four weeks. The rats were euthanized using pentobarbital sodium (ENTOBAR®, 0.5 mL/kg, intraperitoneally; Hanlim Pharm Co., Seoul, Republic of Korea). During the experiment, all the animals had free access to drinking water containing 1% NaCl.

### 2.2. Drug

MGCD0103 was purchased from http://Selleckchem.com (Houston, TX, USA).

### 2.3. Blood Pressure Measurement

The blood pressure of the rats was measured using the tail cuff method for five weeks. The rats were preheated on a hotplate at 35°C for 10 min and then placed in plastic restrainers. A cuff with a pneumatic pulse sensor was attached to the tail. The blood pressure of the rats was recorded using a noninvasive blood pressure controller system (CODA® High Throughput Monitor, Kent Scientific Co., Torrington, CT, USA). The values were presented as the average of at least five consecutive readings obtained from each rat.

### 2.4. Aorta Preparation and Tension Recording

The excised thoracic aorta was placed in a modified Krebs solution containing (in mmol/L) NaCl, 115.0; KCl, 4.7; CaCl_2_, 2.5; MgCl_2_, 1.2; NaHCO_3_, 25.0; KH_2_PO_4_, 1.2; and glucose, 10.0. The connective tissue adhering to the aorta was cleaned on wet filter paper, soaked in Krebs solution, and cut into four ring segments (4.0 mm long). Some rings were denuded of endothelium by gently rubbing the internal surface with the edge of a pair of forceps. Two stainless steel triangles were inserted through each vessel ring. Each aortic ring was suspended in a water-jacketed organ bath (20 mL) maintained at 37°C and aerated with a mixture of 95% O_2_ and 5% CO_2_. One triangle was anchored to a stationary support and the other was connected to an isometric force transducer (Grass FT03C, Quincy, MA, USA). The rings were stretched to an optimal resting tension of 2.0 g, which was maintained throughout the experiment. Each ring was equilibrated in the organ bath solution for 90 min. Then, 50 mmol/L KCl was added, and the contractile response was recorded. Isometric responses were recorded using a computerized data acquisition system (PowerLab/8SP; AD Instruments, Castle Hill, NSW, Australia). Cumulative contractile responses were determined after serial addition of phenylephrine. Cumulative vasorelaxant responses were determined in the aortic rings with or without endothelium by the serial addition of acetylcholine or sodium nitroprusside, respectively.

### 2.5. Histology

The aortic tissues were fixed overnight in 4% formalin, then dehydrated and embedded in paraffin following the conventional method. The paraffin-embedded samples were sectioned into pieces 3 *μ*m thick. The sections were stained with hematoxylin and eosin, trichrome, and Verhoeff-van Gieson staining. For immunohistochemistry, the slides were incubated with alpha smooth muscle actin (*α*SMA, PA5-85070, ThermoFisher Scientific, Waltham, MA, USA), matrix metalloproteinase-2 (MMP2, sc-13594, Santa Cruz Biotechnology, Santa Cruz, CA, USA), and matrix metalloproteinase-9 (MMP9, ab58803, Abcam, Cambridge, UK). The slides were also examined under a light microscope (Axioplan 2 imaging, ZEISS, Jena, Thuringia, Germany), and the slide images were digitalized and transformed into computerized images.

### 2.6. Quantitative Real-Time Polymerase Chain Reaction

Quantitative real-time polymerase chain reaction was performed to detect the expression of *α*SMA (*αSMA*), MMP2 (*Mmp2*), and MMP9 (*Mmp9*) as markers for aortic remodeling, and angiotensin II type 1 receptor (*Agtr1*) and type 2 receptor (*Agtr2*) as markers for angiotensin receptors. The tissues (approximately 100 mg) were homogenized in liquid nitrogen with a glass homogenizer. The RNA was extracted using QIAzol® Lysis Reagent (QIAGEN Science, Germantown, MD, USA) according to the manufacturer's instructions. The total RNA (2 *μ*g) was reverse-transcribed into cDNA using the Thermo Scientific™ RevertAid™ First Strand cDNA Synthesis kit (Fermentas, Vilnius, Lithuania, EU) in a reaction mixture of 20 *μ*L, according to the manufacturer's instructions. Quantitative real-time polymerase chain reaction was performed using the ABI Prism 7000 Sequence Detection System (Applied Biosystems, Foster City, CA, USA). The reaction mixture contained 10 *μ*L of SYBR Green qPCR 2× Master Mix (CellSafe, Yongin-si, Kyounggi-do, Republic of Korea), 4 *μ*L of cDNA, and 200 *μ*L of a primer set (see Table 1). All samples were amplified in triplicate in a 96-well plate under the following cycling conditions: 2 min at 50°C, 10 min at 95°C, and 40 cycles at 95°C for 15 s followed by 1 min at 60°C. The relative mRNA expression level was determined by calculating the values of *Δ* cycle threshold (*Δ*Ct) by normalizing the average Ct value compared with its endogenous control (*Gapdh*), and then calculating the 2^-*ΔΔ*Ct^ value.

### 2.7. Echocardiography

Aortic remodeling was evaluated by echocardiography using a 12 MHz sector array transducer (Philips, Amsterdam, Netherlands). The rats were anesthetized by inhalation of isoflurane gas (1%–3%, Hana Pharm. Co. Ltd., Hwaseong-si, Kyounggi-do, Republic of Korea) during echocardiographic examinations. Two-dimensional color Doppler imaging was used to demonstrate laminar blood flow in the sham group and turbulent blood flow in the TAC group on the aortic arch. Pulsed-wave Doppler was used to measure the velocity of the blood flow in the aortic arch, and the pressure gradient in the arch was calculated using the modified Bernoulli equation (Pressure gradient = 4 × velocity^2^) [21].

### 2.8. Statistics

The results were expressed as mean ± standard error. Kruskal-Wallis test and the one-way analysis of variance (ANOVA), followed by *post hoc* Tukey's comparison test, were performed to analyze the data. Differences were considered significant at *p* < 0.05. Student's *t*-test was applied to analyze the significant differences between two groups. The statistical procedures were performed using SPSS software (version 23.0, SPSS Inc., Chicago, IL, USA).

## 3. Results

### 3.1. MGCD0103 Attenuates Hypertension

**Figure 1:**
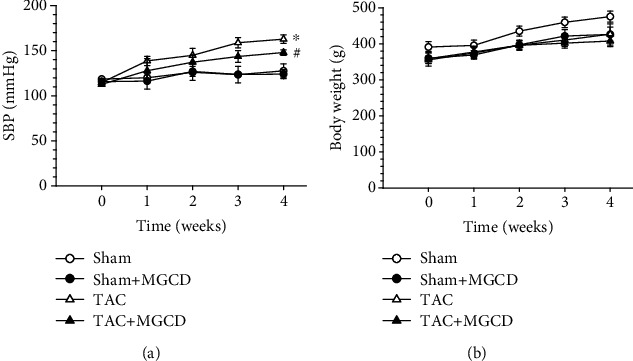
Effect of MGCD0103 (MGCD) on systolic blood pressure and body weight in rats after transverse aortic constriction (TAC). (a) Systolic blood pressure (SBP) was measured using the tail-cuff method in the sham (*n* = 8), sham plus MGCD (*n* = 8), TAC (*n* = 8), and TAC plus MGCD groups (*n* = 8) for five weeks. Administration of MGCD attenuated TAC-induced hypertension. (b) Rat body weights were monitored for four weeks. The body weights of TAC rats treated with or without MGCD were not affected. Data are shown as mean ± standard error of eight rats per group (^∗^*p* < 0.05 vs. sham group, ^#^*p* < 0.05 vs. TAC group).

Systolic blood pressure was measured and recorded for five weeks using the tail-cuff method. TAC resulted in a systolic blood pressure increase of approximately 40 mmHg, which was attenuated to approximately 30 mmHg by the administration of MGCD (Figure 1(a)). Neither TAC nor MGCD administration affected the body weight gain (Figure 1(b)).

### 3.2. MGCD0103 Improves the Vascular Relaxation Response in the Aorta

**Figure 2:**
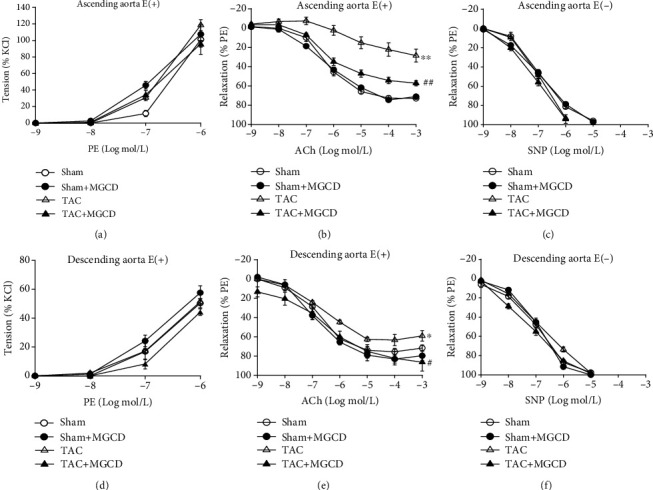
Effects of MGCD on aortic ring contraction and relaxation in rats after TAC. Vascular contractile and relaxant responses were analyzed in the ascending (a–c) and descending (d–f) aortic rings. Phenylephrine (PE) was cumulatively added for vascular contraction in the aortic rings with intact endothelium (a, d) and acetylcholine (Ach) and sodium nitroprusside (SNP) for vasorelaxation in the aortic rings with intact (b, e) and denuded (c, f) endothelia, respectively. The tension developed is expressed as the percentage of maximal contraction to 50 mmol/L KCl, while the relaxation developed is expressed as the percentage of maximal contraction to PE. In the aortic rings with intact endothelium, there was a significant decrease in the relaxation response, and MGCD treatment considerably increased the relaxation response in TAC rats (b, e). Neither TAC nor MGCD affected aortic ring contraction (a, d) or relaxation without endothelium (c, f). Data are shown as mean ± standard error of eight rats per group (^∗^*p* < 0.05 and ^∗∗^*p* < 0.01 vs. sham group, ^#^*p* < 0.05 and ^##^*p* < 0.01 vs. TAC group).

We conducted an organ bath experiment to evaluate whether MGCD affects vascular contraction and relaxation. We investigated the contraction of the aorta by treating it with phenylephrine cumulatively and the relaxation of aorta by treating it with acetylcholine cumulatively. Since shear stress affected different parts of the aorta, especially after TAC, the ascending and descending aorta were evaluated separately in the organ bath experiment. Both the ascending and descending aorta with intact endothelium showed a significant (^∗∗^*p* < 0.01 and ^∗^*p* < 0.05, respectively) decrease in the relaxation response in TAC rats; the MGCD treatment considerably increased the relaxation response in TAC rats (Figures 2(b) and 2(e)), but the TAC and MGCD had greater effects on the ascending aorta. However, neither TAC nor MGCD administration affected vascular contraction (Figures 2(a) and 2(d)) or relaxation in the endothelium-denuded aorta (Figures 2(c) and 2(f)).

### 3.3. MGCD0103 Attenuates TAC-Induced Aortic Remodeling

**Figure 3:**
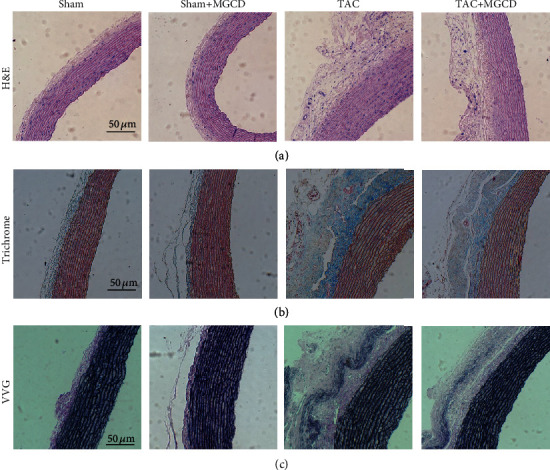
Effect of MGCD on aortic remodeling in rats after TAC. Histological analysis of the aortic wall from the sham (*n* = 8), sham plus MGCD (*n* = 8), TAC (*n* = 8), and TAC plus MGCD groups (*n* = 8) was performed. (a) For hematoxylin and eosin staining (H&E), the thickness of the aortic wall, both in the aortic adventitia and media, was higher in the TAC rats than in the sham rats; the thickness was attenuated by MGCD treatment in both rats. (b) For trichrome staining, fibrosis of the aortic wall, especially in the adventitia (stained blue), was higher in the TAC rats than in the sham rats; the fibrosis was attenuated by MGCD treatment in both rats. (c) For Verhoeff-Van Gieson (VVG) staining, the elastin fibers on the aortic wall, especially in the adventitia (stained dark purple), was higher in the TAC rats than in the sham rats; MGCD treatment attenuated elastin content in the TAC plus MGCD rats. Scale bars are 50 *μ*m.

We performed hematoxylin and eosin staining to confirm aortic hypertrophy histologically in TAC rats. The thickness of the aortic wall, both in the adventitia and the media, was higher in the TAC rats than in the sham rats (Figure 3(a)). Trichrome staining showed fibrosis in the aortic wall, especially in the adventitia. There was more staining in TAC rats compared with that in the sham rats, and MGCD treatment attenuated fibrosis in the TAC plus MGCD rats (Figure 3(b)). We performed Verhoeff-Van Gieson staining to see the change in elastin fibers on the aortic wall. The elastin content in the adventitia was higher in the TAC rats than in the sham rats, and MGCD treatment attenuated elastin content in the TAC plus MGCD rats (Figure 3(c)).

**Figure 4:**
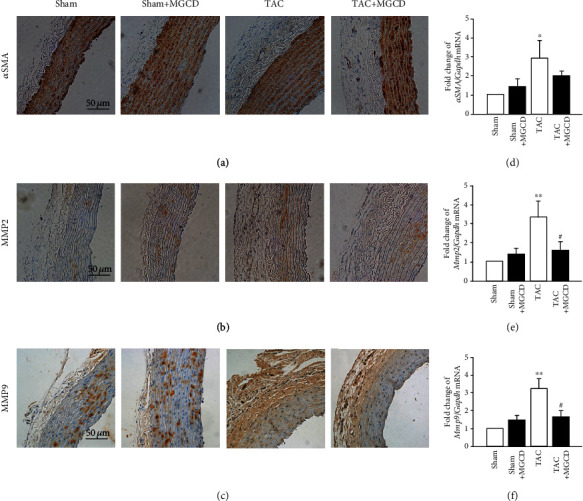
Effect of MGCD on the expression of aortic remodeling markers in rats after TAC. Immunohistochemistry of the aortic wall from the sham (*n* = 8), sham plus MGCD (*n* = 8), TAC (*n* = 8), and TAC plus MGCD groups (*n* = 8) was performed using the anti-*α*SMA (a), anti-MMP2 (b), and anti-MMP9 (c) antibodies. The TAC rats had higher *α*SMA, MMP2, and MMP9 expression levels (brown stain) than the sham rats, but treatment with MGCD decreased their expression in the TAC rats. Scale bars are 50 *μ*m. mRNA expression of *αSMA* (d), *Mmp2* (e), and *Mmp9* (f), markers for myofibroblasts and aortic remodeling, was determined by qRT-PCR. The TAC rats exhibited increased *αSMA*, *Mmp2*, and *Mmp9* mRNA expression, but these decreased with MGCD administration. Data are shown as mean ± standard error of eight rats per group (^∗^*p* < 0.05 and ^∗∗^*p* < 0.01 vs. sham group, ^#^*p* < 0.05 vs. TAC group).

Expression of *α*SMA, a marker for myofibroblasts, was detected by immunohistochemistry. *α*SMA (Figure 4(a)) expression was significantly higher in the TAC rats than in the sham rats, but was attenuated after administering MGCD. We also investigated the expression level of *αSMA* mRNA by qRT-PCR. mRNA expression of *αSMA* (Figure 4(d)) was significantly (^∗^*p* < 0.05) higher in the TAC rats, consistent with the hypertrophy observed during histological evaluation. mRNA expression of *αSMA* decreased with MGCD treatment in TAC plus MGCD rats.

Expression of MMP2 and MMP9, markers for aortic remodeling, was detected by immunohistochemistry. MMP2 (Figure 4(b)) and MMP9 (Figure 4(c)) expression levels were significantly higher in the TAC rats than in the sham rats, and MMP2 and MMP9 were attenuated after MGCD administration. We also investigated *Mmp2* and *Mmp9* mRNA expression levels by qRT-PCR. mRNA expression levels of *Mmp2* (Figure 4(e)) and *Mmp9* (Figure 4(f)) were significantly (^∗∗^*p* < 0.01 and ^∗∗^*p* < 0.01, respectively) higher in the TAC rats than in the sham rats, and MGCD treatment considerably attenuated these transcription levels in the TAC rats. These results were consistent with the changes in fibrosis and elastic fiber observed during the histological evaluation.

### 3.4. MGCD0103 Attenuates the Expression of Angiotensin II Receptor on the Aortic Wall Induced by TAC

**Figure 5:**
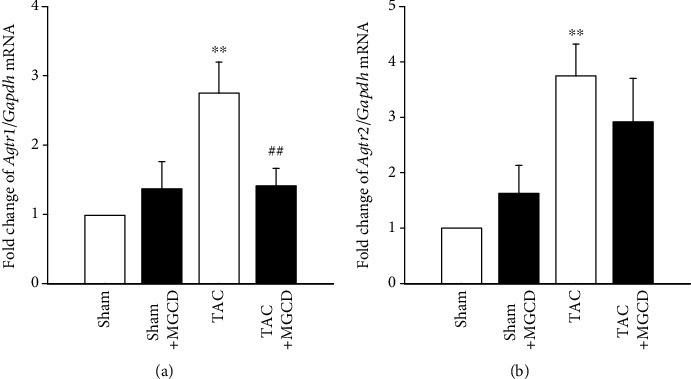
Effect of MGCD on the expression levels of angiotensin II receptors in rats after TAC. mRNA expression levels of angiotensin II type 1 receptor (*Agtr1*) and type 2 receptor (*Agtr2*) were determined by qRT-PCR. The TAC rats had higher *Agtr1* (a) and *Agtr2* (b) mRNA expression, which decreased with MGCD treatment. Data are shown as mean ± standard error of eight rats per group (^∗∗^*p* < 0.01 vs. sham group, ^##^*p* < 0.01 vs. TAC group).

qRT-PCR was performed to determine whether the activation of angiotensin II receptors was regulated by MGCD. *Agtr1* (Figure 5(a)) and *Agtr2* (Figure 5(b)) RNA expressions were significantly higher in the TAC rats than in the sham rats, and MGCD considerably attenuated these transcription levels in the TAC rats.

## 4. Discussion

In the present study, we demonstrated that MGCD, an HDAC inhibitor, attenuates aortic remodeling in rats under pressure overload induced by TAC. Our results showed that MGCD treatment recovered the decreased relaxation response of the aorta with intact endothelium. It also attenuated the aortic remodeling and expression of the angiotensin II receptor on the aortic wall during the pressure overload.

TAC induces a pressure overload via cardiac hypertrophy in rats, and this phenomenon was used as an analogy for hypertensive heart disease in human patients. Starting with the increased pressure on the ascending aortic wall, LV hypertrophy is induced as a compensatory response to hemodynamic pressure overload and eventually leads to LV dysfunction. The organ bath study was performed with the ascending aorta and descending thoracic aorta separately, as the aortic wall and endothelium stress injury may be different [20]. There was a significantly decreased relaxation in the ascending aorta with intact endothelium, and MGCD treatment considerably increased relaxation in the ascending aorta with intact endothelium in TAC rats (Figure 2(b)). TAC and MGCD also elicited a considerable change in the descending thoracic aorta (Figure 2(d)); this change was less than that in the ascending aorta and was not observed during the contraction (Figures 2(a) and 2(d)) or relaxation of the denuded aortas (Figures 2(c) and 2(f)). These results imply that aortic remodeling may obviously start from the ascending aorta due to high pressure on the ascending aorta, which might extend to the descending aorta, and endothelium plays a critical role in aortic relaxation.

During vascular remodeling and fibrosis, collagen, fibronectin, and other extracellular matrix components in the vessel walls are accumulated, which is an important aspect of aortic remodeling in the extracellular matrix during hypertension [17]. Because of the increased pressure on the ascending aorta, shear stress or injury to the endothelium may induce aortic remodeling. Class I HDACs can regulate the morphology of the aorta. MS-275 attenuated the aortic wall thickness and collagen deposition in angiotensin II-infused apolipoprotein E-deficient mice [10], and LMK235 attenuated aortic remodeling in angiotensin II-infused mice and spontaneously hypertensive rats [11]. In the present study, MGCD phenomenologically reduced the systemic blood pressure (Figure 1(a)) and histologically regressed the hypertrophic and fibrotic changes in the aortic wall (Figure 3). Correspondingly, the mRNA expression of myofibroblasts and extracellular matrix remodeling-associated factors (*αSMA*, *Mmp2*, and *Mmp9*) decreased with MGCD treatment (Figure 4). This indicates that the HDAC inhibitor MGCD attenuates aortic remodeling in pressure overload rats under pressure overload.

Angiotensin II is a component of the renin-angiotensin system that plays an important role in cardiovascular physiology by regulating blood pressure and maintaining body water-electrolyte balance. The actions of angiotensin II are mediated by two receptors, angiotensin II type 1 and 2, which are widely expressed in the aortic wall. After TAC, the ascending aorta is under pressure overload, and the increased pulsatile stretch in the ascending aorta may induce mechanical stress on the aortic wall [6, 22]. We investigated the mRNA expression of angiotensin II receptors to determine whether the pressure overload in the aorta alters the vascular expression of angiotensin II receptors, and whether treatment with the HDAC inhibitor regulates this expression. We found that the expression of angiotensin II receptors was related to aortic remodeling; the receptors were expressed by TAC and attenuated by MGCD treatment (Figure 5). Our results are similar to those of previous studies; upregulation of angiotensin II receptors in mouse and rat thoracic aorta by pressure overload [2, 22] and treatment with trichostatin A inhibited angiotensin II activation through angiotensin II receptors [23]. This result suggests that the HDAC inhibitor inhibits angiotensin II signaling, resulting in the attenuation of aortic remodeling.

A few limitations exist in this study. First, the clinical significance of MGCD has not been studied and must therefore be tested in patients which have hypertension-related diseases in further research. Second, the detailed molecular mechanisms of MGCD in regulating aortic remodeling induced by pressure overload is still unknown and should be further explored in future research.

## 5. Conclusions

In summary, the results of this study show that MGCD recovers endothelium dysfunction and aortic remodeling. Moreover, MGCD attenuates the expression of myofibroblasts (*αSMA*) and extracellular matrix remodeling-related (*Mmp2* and *Mmp9)* genes as well as regulates the expression of angiotensin II receptors (*Agtr1* and *Agtr2)* in pressure overload-induced aortic remodeling. Therefore, these findings indicated that MGCD, an HDAC inhibitor, attenuates aortic remodeling in rats with TAC-induced pressure overload rats and may serve as a potential therapeutic target of anti-aortic remodeling in pressure overload-induced hypertension-related diseases.

## Figures and Tables

**Table 1:** Primers for quantitative real-time polymerase chain reaction.

Gene (Accession no.)	Primer sequence (5′ to 3′)	
*α*SMA (NM_031004.2)	F: R:	TCCTGACCCTGAAGTATCCGATA GGTGCCAGATCTTTTCCATGTC
MMP-2 (NM_031054.2)	F: R:	GTGGCAATGGAGATGGACA GGTCATAATCCTCGGTGGTG
MMP-9 (NM_031055.1)	F: R:	AGGCGCCGTGGTCCCCACTTACTT GCAGGGTTTGCCGTCTCCGTTGCC
Agtr1 (XM_008771594.2)	F: R:	GGAGAGGATTCGTGGCTTGAG CTTTCTGGGAGGGTTGTGTGAT
Agtr2 (NM_012494.3)	F: R:	CATCACCAGCAGTCTTCCTTTTG AAAACAGTGAGACCACAACAATGT

## Data Availability

The data used to support the findings of this study are included within the article.
